# Fitness Effects of 10-Month Frequent Low-Volume Ball Game Training or Interval Running for 8–10-Year-Old School Children

**DOI:** 10.1155/2017/2719752

**Published:** 2017-02-19

**Authors:** Malte Nejst Larsen, Claus Malta Nielsen, Christina Ørntoft, Morten Bredsgaard Randers, Eva Wulff Helge, Mads Madsen, Vibeke Manniche, Lone Hansen, Peter Riis Hansen, Jens Bangsbo, Peter Krustrup

**Affiliations:** ^1^Department of Sports Science and Clinical Biomechanics, SDU Sport and Health Sciences Cluster (SHSC), University of Southern Denmark, Odense, Denmark; ^2^Department of Nutrition, Exercise and Sports, Copenhagen Centre for Team Sport and Health, University of Copenhagen, Copenhagen, Denmark; ^3^Health Frederikssund Municipality, Frederikssund, Denmark; ^4^LIVA Group, Holte, Denmark; ^5^Team Denmark, Brøndby, Denmark; ^6^Department of Cardiology, Herlev-Gentofte University Hospital, Gentofte, Denmark; ^7^Sport and Health Sciences, College of Life and Environmental Sciences, University of Exeter, Exeter, UK

## Abstract

We investigated the exercise intensity and fitness effects of frequent school-based low-volume high-intensity training for 10 months in 8–10-year-old children. 239 Danish 3rd-grade school children from four schools were cluster-randomised into a control group (CON, *n* = 116) or two training groups performing either 5 × 12 min/wk small-sided football plus other ball games (SSG, *n* = 62) or interval running (IR, *n* = 61). Whole-body DXA scans, flamingo balance, standing long-jump, 20 m sprint, and Yo-Yo IR1 children's tests (YYIR1C) were performed before and after the intervention. Mean running velocity was higher (*p* < 0.05) in SSG than in IR (0.88 ± 0.14 versus 0.63 ± 0.20 m/s), while more time (*p* < 0.05) was spent in the highest player load zone (>2; 5.6 ± 3.4 versus 3.7 ± 3.4%) and highest HR zone (>90% HR_max_; 12.4 ± 8.9 versus 8.4 ± 8.0%) in IR compared to SSG. After 10 months, no significant between-group differences were observed for YYIR1C performance and HR after 2 min of YYIR1C (HR_submax_), but median-split analyses showed that HR_submax_ was reduced (*p* < 0.05) in both training groups compared to CON for those with the lowest aerobic fitness (SSG versus CON: 3.2%  HR_max_ [95% CI: 0.8–5.5]; IR versus CON: 2.6%  HR_max_ [95% CI: 1.1–5.2]). After 10 months, IR had improved (*p* < 0.05) 20 m sprint performance (IR versus CON: 154 ms [95% CI: 61–241]). No between-group differences (*p* > 0.05) were observed for whole-body or leg aBMD, lean mass, postural balance, or jump length. In conclusion, frequent low-volume ball games and interval running can be conducted over a full school year with high intensity rate but has limited positive fitness effects in 8–10-year-old children.

## 1. Introduction

It is well known that regular physical activity plays a crucial role in preventing a number of diseases challenging modern society [1], and studies show that children who are involved in regular activities in sports clubs have better aerobic fitness and higher bone mineralisation than children who are not members of local clubs [2–4]. Most recommendations on physical activity in childhood, to which school-based activities contribute, focus on training duration, for example, 60 min/day [5, 6], as recommended by WHO. However, recent evidence has shown that intense training is more effective for adults and adolescents in terms of improving musculoskeletal, metabolic, and cardiovascular fitness compared to moderate-intensity training [7–10]. In relation to children, recent studies have shown that the intensity during various small-sided games (SSG), such as football, basketball, and hockey, is high [11, 12]. In large Danish and international school-based interventions, which have attempted to increase physical activity in children for health improvement, the duration of each physical activity session has often been prolonged, for example, 60 min [13]. If schools are to contribute to children's health, a time-saving and potentially health-promoting model is desirable. However, such a model has not yet been scientifically tested.

Bone mass is built up during growth through childhood and adolescence, and peak bone mass appears to be crucial for bone health in adults and for reducing the risk of osteoporosis later in life [14–16]. Studies have shown that both boys and girls involved in team sports have greater bone mineral content (BMC) and bone density (BMD) than children who are not engaged in sports, as well as children who only participate in non-weight-bearing sports, leading to low impact on bones, such as cycling and swimming [17–20]. Also aerobic fitness levels in children are of great importance, since low aerobic fitness and obesity to a large extent track from childhood to adulthood [21]. There is evidence that interval running training has positive cardiovascular effects for children and adolescents [22, 23], but it may have limited musculoskeletal effects, as has been suggested in adults [10]. However, in adults, football has positive cardiovascular effects [24] as well as positive musculoskeletal effects [25, 26], and football is superior to interval running training to achieve such effects [10, 26]. For children, results from our large-scale randomised Frequent Intense Training—Football, Interval Running and Strength Training study (the FIT FIRST RCT) showed that 3 × 40 min/wk of small-sided ball game training over 10 months in 8–10-year-old children resulted in broad-spectrum musculoskeletal improvements, including increases in muscle strength, postural balance, and bone mineralisation [27]. It is unclear, however, whether more frequent but lower-volume training at school can have similar favourable effects.

Therefore, the aim of the present study was to investigate health-related fitness effects of frequent school-based low-volume high-intensity training over 10 months for 8–10-year-old children using 5 × 12 min per week of ball games or interval running.

## 2. Methods

A total of 239 Danish 8–10-year-old 3rd-grade school children from four schools were cluster-randomised to SSG (*n* = 62, 3 classes), IR (*n* = 61, 3 classes), or a control group (CON, *n* = 116, 5 classes). 66% of these children were regularly active in leisure-time sports clubs (71% in SSG, 67% in IR, and 60% in CG). Two rounds of cluster randomisation were used to assign one control school and two intervention schools in the two geographical areas (rural or urban) and to assign the two or three classes in the intervention schools to SSG or IR, with both training types represented at all intervention schools. Two staff members filled sets of identical, sealed envelopes with the names of the schools and school classes, respectively, along with two sets of envelopes that contained the numbers 1-2, 3-4, and 5-6. A third researcher blinded to the filling of the envelopes chose the envelopes and rolled a dice to decide group allocation and training type, respectively.

All pretests and posttests were performed during school time at the beginning and end of the school year by university staff members with the support of school teachers. Written informed parental consent was obtained for all participants. The study was approved by the Committees on Biomedical Research Ethics for the Capital Region of Denmark (J.no. H-3-2013-038). The present study is part of the large-scale FIT FIRST study that includes a total of more than 400 children (ClinicalTrials.gov: NCT02000492). The current study focused on the effects of 5 × 12 min/wk (1 hr/wk) of SSG and IR training versus a control group, whereas data for the two training groups comprising medium-frequency low-volume (3 × 40 min/wk) SSG and CST has been and will be presented in future publications [27].

All the children were interviewed and examined by a medical doctor (CMN) who also assessed individual Tanner stages. Two children were excluded due to physical disabilities. During the interview, the children were, among other questions, asked whether they participated in organised sporting activities at least once a week (yes or no) (Table 1).

### 2.1. Training Intervention

Both types of training were led by trainers engaged by the university and performed at the schools every morning for 12 min/session. The team sports consisted mainly of 3v3 football, basketball, and unihockey, which have all been shown to have high involvement and exercise intensity for all children [11]. For practical reasons, other SSGs were occasionally used, and, depending on the weather, both outdoor and indoor facilities were used.

The IR was conducted outdoors on grass or asphalt. The children ran in high-intensity intervals of 1 min with 30 s rest periods in between.

### 2.2. Anthropometric Measures and Body Composition

The children were weighed barefooted and wearing light clothing (Tanita WB-110MA, Tanita, Europe) and their height was measured (235 Heightronic Digital Stadiometer, QuickMedical, Issaquah, WA, US). A whole-body (wb) DXA scan (Lunar Prodigy; E Medical Systems, Madison, Wisconsin, USA) using Encore software version 13.5 (Encore, Madison, USA) was performed in accordance to standard procedures to estimate whole-body and leg aBMD and BMC as well as lean body mass. The children were scanned in a supine position. The manufacturer states a coefficient of variation (CV) for whole-body bone variables less than 1%. This is supported by a study that evaluated the leg region in children from a whole-body scan and reported CV values of 1.11 to 1.36% for regional bone parameters [28]. For each child, pretesting and posttesting were conducted at the same time of day (within one hour) and the children were instructed to fast for at least 2 hr and to visit the toilet before the scan.

### 2.3. Yo-Yo Intermittent Recovery Level 1 Children's Test (YYIR1C)

After a standardized warm-up (5 min of low to high speed running, arm swinging, and jumping), including the first three 2 × 16 m shuttle runs of the test, YYIR1C, a modified version of the Yo-Yo intermittent recovery level 1 test [29], was used to determine the aerobic fitness level. The test was carried out at the schools in indoor sports facilities. The subjects were instructed to run 16 m back and forth in a straight lane marked by cones, followed by a 10 s recovery period in which they walked around a cone 4 m behind the running lane. The running pace was set by beep signals and increased gradually during the test according to the test protocol [29]. The first time the subject failed to complete the running bout in due time, a warning was issued. In the second time, the test was terminated and the result was noted by the research team. One researcher also participated in the test to show the pace to the children and to motivate the last children running. During the tests, heart rate (HR) was recorded at 1 s intervals using a Polar Team System 2 (Polar Electro Oy, Kempele, Finland). To determine maximal HR (HR_max_), the highest value during each test was registered. Submaximal heart rate (HR_submax_) was determined 2 min into the test as mean HR from 1 : 45 to 2 : 15 min. HR_submax_ and YYIR1C performance have been shown to be valid measures of aerobic fitness in children below 10 yrs [29, 30].

### 2.4. Postural Balance

Postural balance was evaluated using the single-leg flamingo balance test [31] performed on a 3 cm wide, 5 cm high, and 50 cm long balance beam. The subject was instructed to stand barefooted on one leg on the bar, with eyes open, holding the contralateral leg at the ankle joint. The number of times the subject fell off the bar (defined as touching the ground and/or unable to hold the contralateral leg at the ankle joint) before completing 1 min balance in total was counted and used as an indicator of postural balance [31]. The subjects had one try with each leg in order to choose their preferred leg for the test. If 20 falls were counted before 1 min had passed, the time of the final fall was noted and used to calculate the expected number of falls during a full 1-minute period. The test is reliable and valid as a test for balance in young children [32].

### 2.5. Maximal Horizontal Jump Length

After warm-up, including two submaximal jumps, maximal horizontal jump performance was determined from (1) a squat jump (SJ) with arms held steady behind the back in an upright standing position, where the child flexed the knees to the squat position, held that position for 2 s, and then jumped forward, and (2) a countermovement jump (CMJ) following the same procedure but allowing use of the arms. After the standardised warm-up, the children were placed behind a line with their feet parallel and shoulder-width apart. They first performed the CMJ and then the SJ after a 2-minute rest period. In both jumps, distance from the starting line to the heel closed to the line was measured. This is a reliable method [33] and maximal horizontal jump performance is well correlated to leg muscle strength in children [34, 35].

### 2.6. 20 m Sprint Test

After warm-up, including high speed running, the children performed 2 × 20 m maximal sprints with at least 2 min of recovery between sprints. All sprints started from a standing position and were timed using two ports of photocells (Witty Microgate, Bolzano, Italy) placed at 0 m (positioned 30 cm in front of the standing start position) and at 20 m. The test result was the fastest time recorded [33]. Sprinting ability depends on muscle strength and power, body weight, and neuromuscular function.

### 2.7. Coordination

Time to complete a coordination wall with three stages of increased difficulty was used to evaluate coordination abilities [36]. Each stage consisted of a table (9  ×  8 A5 size squares) with numbered marks from 1 to 10. Five of the numbers were blue and five were red. The two lowest ranks were separated from the upper ranks by a thick line. The subjects had a red mark on the right hand and a blue mark on the left hand and were instructed to touch the numbers from one to ten in numeric order and as fast as possible with the hand (above the thick line) or the foot (below the thick line) matching the colour of the number. The participants were instructed to correct the mistake and proceed if they made such, while the clock was still ticking. Stage 1 and stage 2 were unilateral; stage 1 was with no crossing over the vertical midline and stage 2 was with crossing over the vertical midline. Stage 3 was bilateral with both colours on both sides of the wall. The participants were given three attempts at each stage and the shortest times for each stage were summed to provide a combined score for stages 1, 2, and 3.

### 2.8. Heart Rate Monitoring

Heart rate was recorded beat by beat in 1 s intervals during training using short-range radio telemetry (Polar Team 2 System, Polar Electro Oy, Kempele, Finland).

### 2.9. Player Load and Running Velocity

The activity profile for each participant during training was measured using a portable GPS and accelerometer device (MinimaxX S4, Catapult Innovations, Canberra, Australia). The GPS unit was placed in a harness designed by the manufacturer, which was worn during training. After recording, the data was downloaded and analysed using the Catapult Sprint software (Catapult Sports, Canberra, Australia). Running velocity was analysed as means as well as percentage of distance covered at low (0–2 m/s), medium (2–4 m/s), and high speed (>4 m/s). Player load is an estimate of training intensity combining the rate of change in acceleration in three planes (forward [fwd], sideways [side], and upwards [up]) using the following formula:

(1) $$\begin{array}{c} \sqrt{\left({\left({fwd}_{t=i+1}-{fwd}_{r=i}\right)}^{2}+{\left({side}_{t=i+1}-{side}_{t=i}\right)}^{2}+{\left({up}_{t=i+1}-{up}_{r=i}\right)}^{2}\right)}. \end{array}$$

Player load has been proven to be highly correlated with Edwards' and session-RPE methods [37] and has shown high reliability, suggesting that accelerometers may be used to determine the physical demands of various types of high-intensity training [38]. The percentage of training time in different player load zones (0-1 (low), 1-2 (medium), and >2 (high)), as well as mean values, was obtained from the software.

### 2.10. Statistics

Unless otherwise stated, data are presented as means ± SD. Baseline values for the three intervention groups (CON, SSG, and IR) were compared using one-way analysis of variance. To evaluate the intervention-induced effects, change scores from 0 to 10 months for CON, SSG, and IR were compared using one-way analysis of variance with SAS Enterprise Guide 7.1 (SAS institute Inc., Cary, NC, USA). Only data sets with complete preintervention and postintervention values were included in the analysis. Between-group differences in training intensity were tested using one-way analysis of variance. The significance level was set at 0.05.

## 3. Results

No between-group differences were observed in height, body mass, BMD, BMC, lean mass, and physical capacity at baseline (Tables 1 and 2). During the 10-month intervention period, no differences were observed in the change in height and body mass between groups. Only a few minor injuries (bruises and strains) and no major injuries were observed during the study period with no apparent difference between training groups.

### 3.1. Intensity During Training

**Figure 1:**
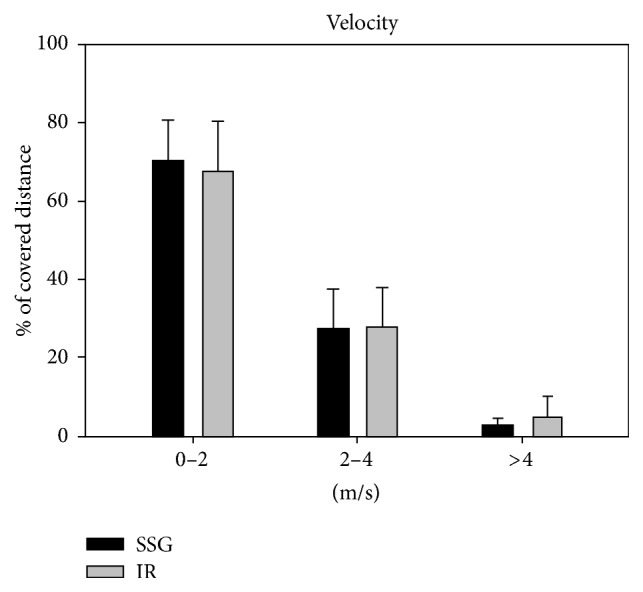
Fraction of distance covered in various speed zones during low-volume small-sided games (SSG, black bars) and interval running training (IR, grey bars). Data are presented as means ± SD.

**Figure 2:**
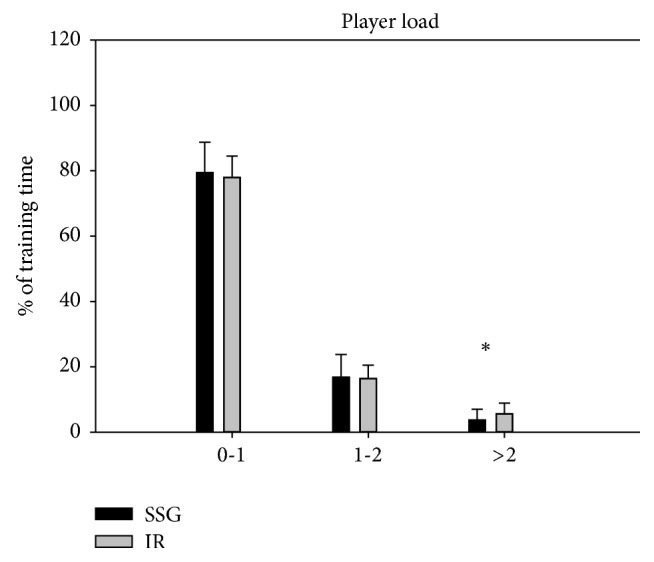
Time spent (%) in various player load zones (0-1 (low), 1-2 (medium), and >2 (high) specified in the accelerometers manufacturer's software) during low-volume small-sided games (SSG, black bars) and interval running training (IR, grey bars). Data are presented as means ± SD. *∗* denotes significant difference between SSG and IR at *p* < 0.05.

**Figure 3:**
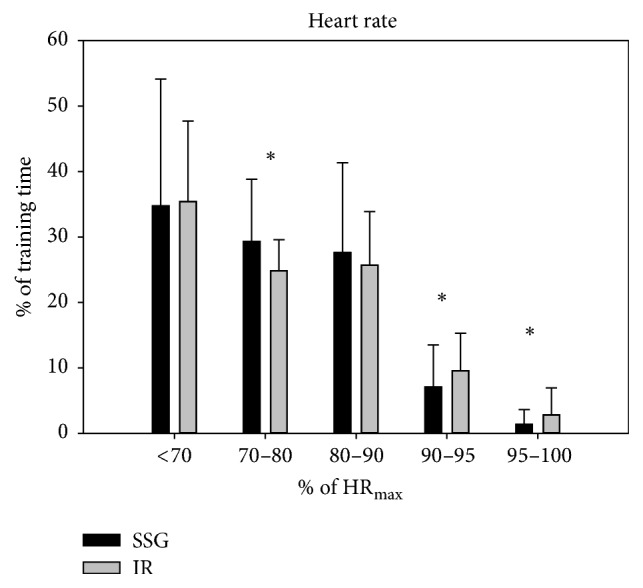
Time spent (%) in various heart rate zones during low-volume small-sided games (SSG, black bars) and interval running training (IR, grey bars). Data are presented as means ± SD. *∗* denotes significant difference between SSG and IR at *p* < 0.05.

Mean velocity during training was higher (*p* < 0.05) in SSG than in IR (0.88 ± 0.14 versus 0.63 ± 0.20 m/s), while more time (*p* < 0.05) was spent in the highest (>2) player load zone (5.6 ± 3.4 versus 3.7 ± 3.4%, *p* < 0.05) during IR compared to during SSG (Figures 1 and 2). Participants spent more time in the heart rate zone of 70–80%  HR_max_ (*p* < 0.05) during SSG than during IR (29.3 ± 9.5 versus 24.8 ± 4.8%), whereas time spent above 90%  HR_max_ was higher (*p* < 0.05) during IR (12.4 ± 8.9 versus 8.4 ± 8.0%) (Figure 3). No differences were observed in mean player load (SSG: 0.58 ± 0.17; IR: 0.56 ± 0.13) and heart rate (SSG: 73.9 ± 5.3%  HR_max_; IR: 73.9 ± 3.8%  HR_max_) nor percentage of distance covered at the highest (>4 m/s) velocities (SSG: 2.5 ± 1.9%; IR: 4.7 ± 4.7%) (Figures 1–3).

### 3.2. Fitness Effects

After 10 months, IR had positive effects (*p* < 0.05) on 20 m sprint performance (IR versus CON: 154 ms [95% CI: 61–241 ms]). After 10 months, no significant between-group differences were observed for YYIR1C performance and HR after 2 min of YYIR1C (HR_submax_), but median-split analyses showed that HR_submax_ was reduced (*p* < 0.05) in both training groups compared to CON for those with the lowest aerobic fitness (SSG versus CON: 3.2%  HR_max_ [95% CI: 0.8–5.5]; IR versus CON: 2.6%  HR_max_ [95% CI: 1.1–5.2]).

No between-group differences were observed for whole-body or leg aBMD, BMC, lean body mass, postural balance, gross motor skill, YYIR1C performance, and jump length, respectively (all *p* > 0.05) (Table 2).

## 4. Discussion

The main findings of the present study were that brief daily sessions of small-sided ball games (SSG) and interval running (IR) can be conducted with high intensity and attendance for 8–10-year-old school children. It was also shown that a period of 10 months with five weekly 12-minute sessions of IR training resulted in increases in sprint performance and that both SSG and IR training lowered heart rate during standardised submaximal interval exercise for those that had the poorest baseline values, indicating that increased cardiovascular fitness was achieved in these subjects. No significant training effects were observed in musculoskeletal fitness.

In the present study, we found that the average speed during small-sided ball game training was as high as for 9-year-old boys participating in 20-minute long recreational 5v5 and 8v8 football match-play [39] and that the player loads were relatively high during the small-sided ball game training with values around two-thirds of the values seen in the 5v5 football matches [39]. With regard to IR, the average running speed was 30% lower than for the small-sided ball game training, but the time in the highest player load zone was actually 50% higher than in SSG. These high player loads in IR are primarily related to intense accelerations in the beginning of each running interval. The children were typically eager to get going but rarely able to maintain the running speed towards the end of each running bout. This type of running performed by the IR group led to better sprinting ability after 10 months of daily training. Considering the lack of effects of training on jump length and body weight observed in the current study, it may be speculated that the improvements in sprinting performance relate to improvements in coordination and anaerobic power rather than leg muscle strength.

The frequent low-volume SSG and IR were not sufficient to improve the bone mineralisation and muscular fitness of 8–10-year-old school children despite the fact that the player loads were similar to those found in another FIT FIRST study that examined effects of medium-volume interventions (3 × 40 min SSG or circuit strength training) [27]. In the latter study, marked improvements were observed in jump length, sprint performance, and bone mineralisation over the same 10-month intervention period [27]. The explanations for this difference may be the bigger training volume and the fact that the sessions in the latter study were more than 3 times longer (40 min versus 12 min), so these children spent more time in the fatigued state, which may be a major factor contributing to higher bone strains, since under such circumstances muscle ability to absorb shocks is lower [40]. Bone strains are difficult to determine in humans, whereas it is possible to measure the ground reaction forces and, as done in the present study, the player load, which can provide indirect information about the skeletal loading and the resulting strains. Other studies have reported site-specific changes in aBMD and BMC in prepubertal boys and girls with training volumes as low as 3 × 10 min/wk [41–45] involving high-impact exercises such as jumping, indicating that it is possible to induce bone mineralisation with even lower training volumes than those investigated in the present study. Taken together, this may indicate that bone health-related (osteogenic) responses to exercise depend not only on the strain magnitude and rates induced by training but also on exercise duration in each of the training sessions and the overall training volume, respectively.

The heart rate recordings showed that the average HR was 74%  HR_max_ in SSG and IR with about 40% of the training time with HR above 80%  HR_max_ and 8.4 and 12.4%, respectively, of the time in the highest aerobic training zone above 90%  HR_max_. These findings clearly show that brief sessions with SSG as well as IR could be performed with high average intensity and with periods of near-maximal aerobic loading. Previous studies have shown marked positive effects on YYIR1C performance for 8–10-year old school children of 2 × 30 min of weekly training with a comparable heart rate response [11]. Thus, large improvements in both maximal and submaximal YYIR1C performances were reported after 6 weeks of SSG, whereas the control group performing low-to-moderate PE activities had no effects on YYIR1C performance or submaximal HR scores during the YYIR1C. In the present study, we analysed the between-group differences in change scores over the full 10-month period, and no significant differences were observed in the changes between training groups and the control group. However, median-split analysis focusing on the subjects with the poorest baseline level revealed positive effects on HR_submax_, indicating that the former group can achieve improved fitness by frequent low-volume exercise interventions. On one hand, these findings indicate that frequent brief intense training sessions may not have a very pronounced effect on intermittent exercise performance and aerobic fitness for the average Danish 8–10-year-old school child, but, on the other hand, a high attendance rate and positive training effects were observed for those children with the lowest aerobic fitness, suggesting that these time-efficient training types are worth considering when planning PE sessions aiming at improving aerobic fitness of those that need it the most.

Interestingly, few injuries and no major injuries were observed in the present study. Prior to the study, some parents and teachers were nervous about the risk of injuries when playing football, but our findings align with studies showing a low injury risk during training sessions for 10-year-old children, since injury frequency is only about 5 injuries per 1,000 hours of soccer practice [46]. Also, a recent study did not report injuries after a school-based football intervention involving SSG and skill-developing drills [47]. To our knowledge, no previous studies have examined the injury risk from running in children [48] but the present results clearly suggest that school-based low-volume SSG and IR are safe for 8–10-year-old children.

Motivation is an important aspect when developing best-practice physical activity interventions. As a part of the present study, motivation was shown to be lowered for IR during the intervention period, whereas the motivation was kept constant for the SSG group [49], indicating that the SSG intervention has greater potential for long-term implementation.

In summary, the overall fitness effects of 10 months of intense frequent low-volume training was limited, but positive effects were seen in sprint performance in the interval running group and in cardiovascular strain in the ball game group, as well as in the interval running group. Furthermore, the present study revealed that frequent low-volume ball games and interval running can be conducted over a full school year with high intensity and high attendance rate.

### 4.1. Perspectives

This time-effective training concept has potential health benefits and can easily be conducted and be a part of the total amount of physical activity that children do every day, alongside activities such as active transportation, PE lessons, games during break time, and leisure-time sport. Future reports from the FIT FIRST study will examine the effects of 12 min of daily training on cardiovascular health, psychosocial and cognitive function, and scholastic performance, which should be taken into account in aggregate when interpreting the overall health-promoting effects of the intervention, and studies combining the training with other types of physical activity and/or nutrition could be of future interest.

## Figures and Tables

**Table 1:** Subjects characteristics in relation to group (SSG, IR, and CON) and gender before (pre) and after (post) the 10-month intervention period. Data are presented as means ± SD, except for Tanner stage distribution (% in 1/2/3, resp.) and percentage of children active in sports clubs (% of all).

		SSG		IR		CON	
		Pre	Post	Pre	Post	Pre	Post
Age (yrs)	All Boys Girls	9.4 (±0.4) 9.3 (±0.3) 9.4 (±0.4)	10.1 (±0.4) 10.0 (±0.3) 10.1 (±0.4)	9.3 (±0.3) 9.3 (±0.3) 9.4 (±0.3)	10.0 (±0.3) 10.0 (±0.3) 10.0 (±0.3)	9.3 (±0.3) 9.3 (±0.3) 9.4 (±0.3)	10.0 (±0.4) 10.0 (±0.3) 10.0 (±0.4)

Weight (kg)	All Boys Girls	31.8 (±4.4) 31.6 (±4.6) 32.1 (±4.2)	34.4 (±4.8) 34.0 (±4.8) 34.9 (±4.9)	32.7 (±5.6) 33.5 (±5.9) 31.6 (±5.2)	35.0 (±6.0) 35.9 (±6.1) 33.7 (±5.7)	32.8 (±6.0) 31.7 (±5.2) 33.5 (±6.7)	35.2 (±6.8) 33.8 (±5.1) 36.3 (±7.8)

Height (cm)	All Boys Girls	138.8 (±5.0) 139.3 (±4.7) 138.0 (±5.4)	142.5 (±5.3) 142.9 (±4.9) 142.0 (±5.9)	139.8 (±6.0) 141.1 (±5.6) 138.2 (±6.2)	143.3 (±6.0) 144.4 (±5.6) 141.8 (±6.3)	138.4 (±6.0) 137.9 (±5.3) 138.7 (±6.5)	142.2 (±6.4) 141.5 (±5.3) 142.8 (±7.0)

Tanner stage (1/2/3) %	All Boys Girls	86/14/0 100/0/0 68/32/0	81/19/0 100/0/0 56/44/0	98/2/0 100/0/0 96/4/0	93/7/0 100/0/0 83/17/0	84/14/2 100/0/0 70/26/3	73/27/0 100/0/0 54/46/0

Sports club active (%)	All Boys Girls	71% 74% 69%	72% 74% 70%	67% 72% 63%	67% 72% 63%	60% 59% 61%	61% 59% 59%

**Table 2:** Body composition and functional capacity measurements for the control group (CON) and the two intervention groups performing 5 × 12 min per week of small-sided ball games (SSG) and interval running (IR) before (pre) and after (post) the 10-month intervention period.

	SSG		IR		CON	
	Pre	Post	Pre	10 months	Pre	10 months
Balance (falls/min)	19.6 (±8.1)	18.0 (±7.0)	20.4 (±8.2)	18.5 (±6.0)	20.8 (±8.5)	20.7 (±7.7)
Jump distance (% of pre)	100	104 (±12)	100	103 (±12)	100	100 (±12)
20 m sprint (s)	4.24 (±0.34)	4.22 (±0.32)	4.48 (±0.33)	4.23 (±0.24)^*∗*^	4.41 (±0.34)	4.31 (±0.33)
Coordination wall time (s)	66 (±10)	52 (±11)	66 (±12)	56 (±10)	67 (±16)	57 (±11)
YYIR1C (m)	682 (±450)	845 (±490)	647 (±450)	820 (±480)	712 (±411)	838 (±447)
YYIR1C_submax_ (% of HR_max_)	91.3 (±4.5)	89.6 (±4.2)	91.1 (±4.4)	88.5 (±4.1)	91.2 (±4.5)	90.0 (±4.4)
BMD (g/cm^2^)						
Whole body	0.89 (±0.05)	0.91 (±0.06)	0.90 (±0.05)	0.92 (±0.05)	0.88 (±0.05)	0.90 (±0.05)
Leg	0.87 (±0.07)	0.91 (±0.08)	0.88 (±0.07)	0.92 (±0.07)	0.86 (±0.07)	0.90 (±0.08)
BMC (g)						
Whole body	1173 (±166)	1281 (±181)	1220 (±164)	1316 (±177)	1169 (±186)	1277 (±204)
Leg	405 (±70)	456 (±80)	421 (±75)	473 (±85)	400 (±82)	453 (±93)
Lean mass (kg)						
Whole body	23.00 (±2.58)	24.79 (±2.90)	24.10 (±2.69)	25.50 (±2.77)	23.39 (±2.64)	25.02 (±2.88)
Leg	7.55 (±0.94)	8.15 (±1.13)	7.99 (±1.04)	8.65 (±1.10)	7.64 (±0.99)	8.37 (±1.15)

Data are presented as means ± SD. *∗* denotes difference in change scores compared to CON; *p* < 0.05.

BMD, bone mineral density; BMC, bone mineral content; YYIR1C, Yo-Yo Intermittent Recovery Level 1 Children's test.

## Abbreviations

ANOVA: Analysis of variance
BMC: Bone mineral content
aBMD: Areal BMD (g/cm^2^)
BMD: Bone mineral density
CI: Confidence Interval
DXA: Dual energy X-ray absorptiometry
FIT FIRST: Frequent Intense Training—Football, Interval Running and Strength Training
HR: Heart rate
IR: Interval running
LBM: Lean body mass (kg)
SD: Standard deviation
SSG: Small-sided games
YYIR1C: Yo-Yo Intermittent Recovery Level 1 Children's test.
